# Task shifting interpersonal counseling for depression: a pragmatic randomized controlled trial in primary care

**DOI:** 10.1186/s12888-017-1379-y

**Published:** 2017-06-21

**Authors:** Camila T. Matsuzaka, Milton Wainberg, Andrea Norcini Pala, Elis V. Hoffmann, Bruno M. Coimbra, Rosaly F. Braga, Annika C. Sweetland, Marcelo F. Mello

**Affiliations:** 1 Department of Psychiatry, Federal University of São Paulo (UNIFESP), R. Borges Lagoa 570/10 andar, 04038-000 São Paulo, SP Brazil; 2 Division of Epidemiology New York State Psychiatric Institute, New York, NY USA; 3 Department of Psychiatry, Columbia University College of Physician and Surgeons, New York, NY USA; 4 HIV Center for Clinical and Behavioral Studies New York State Psychiatric Institute, New York, NY USA

**Keywords:** Depression, Primary care, Interpersonal counseling, Randomized controlled trial, Low and middle-income countries

## Abstract

**Background:**

Task shifting approaches (rational redistribution of tasks among health workforce teams) to train lay professionals to assist with integrating mental health treatment in primary care has been recommended to close the mental health treatment gap for depression in low- and middle-income countries. This study aims to examine the a new model for depression care in a low-resource environment compared to enhanced treatment at usual (E-TAU).

**Methods:**

We trained non-specialist community health workers (local lay employees of the public health system) to provide Interpersonal Counseling (IPC) to treat depressive symptoms in the Brazilian, São Paulo city, family health strategy (FHS). We conducted a randomized controlled trial involving 86 patients with a current major depressive disorder or dysthymia (based on DSM-IV) recruited from an FHS clinic. Participants were randomized to IPC intervention (*n* = 43) or E-TAU (*n* = 43). Participants allocated to IPC received 3–4 sessions provided by community health workers; research psychologists followed the E-TAU participants to facilitate their referral to specialized mental health care within the public system. Reduction of depressive symptoms was assessed using the Hamilton Rating Scale (HDRS-17) and the Patient Health Questionnaire (PHQ-9); minor psychiatric symptomatology (including depression, anxiety and somatoform symptoms) were measured using the Self Reporting Questionnaire (SRQ); and functioning was measured by the Clinical Global Impression Scale over a 2-month period.

**Results:**

Intention-to-treat analysis showed significant improvement on symptoms for both groups over 2 months, without significant differences between them. Per-protocol analysis showed significant better HDRS-17 outcomes for the IPC group.

**Conclusions:**

Training non-specialist community health workers in low- and middle-income countries to provide IPC could be a successful strategy in reducing the burden of depression and also potentially a low-cost and effective alternative to specialist-led services that might not be possible in low income settings.

**Trial registration:**

Brazilian Clinical Trials, number RBR-5qhmb5 (trial url: http://www.ensaiosclinicos.gov.br/rg/RBR-5qhmb5/), retrospectively registered after May 1, 2013.

## Background

The World Health Organization (WHO) ranks Major Depressive Disorder (MDD) as one of the most significant challenges for the twenty-first century, because of its consequent disability and loss of function. MDD is the leading cause of neuropsychiatric burden of disease globally [1, 2]. The majority of people with MDD can be treated early and effectively in primary care [3]; nevertheless MDD is underdiagnosed and undertreated [4–6]. The global mental health treatment gap is more pronounced in low and middle-income countries (LMIC), where the vast majority of mental health needs is unmet [7]. There is a large literature supporting psychological non-pharmaceutical interventions for treating MDD, which are often preferred by primary care patients [8–10] and are recommended as first-line treatment by the international guidelines [11–13].

Brazil’s Family Health Strategy (FHS) has made remarkable progress towards universal coverage of its population in primary care in the last decades, but only limited investments in mental health [14]. Specialized community-based psychosocial care centers were established to provide treatment to individuals with severe mental illness [15] but no strategies were developed to integrate the treatment of common mental disorders within primary care. Community health workers are employees of the public health system; each one is assigned to approximately 150 households within the catchment area of a given FHS outreach clinic. They visit each household at least once per month and gather information about health-promotion activities and basic clinical care [14]. They are also tasked with identifying potential warning signs of violence, neglect, truancy or drug use, but receive no mental health training [16–18]. Community health workers are therefore an important human resource for health in Brazil. Globally, there is an increased focus on the use of lay community health workers to meet mental health needs [19–21].

Interpersonal psychotherapy (IPT) is an evidence-based intervention [13, 22] that has been effectively implemented in low income settings using a task-shifting approach, wherein non-specialist lay counselors are trained to deliver the intervention with expert supervision [23]. The WHO defines task shifting as “the rational redistribution of tasks among health workforce teams” [19]. In other words, specific functions are shifted, where appropriate, from highly qualified health workers to health workers with shorter training and fewer qualifications in order to make more efficient use of the available human resources for health.

Interpersonal counseling (IPC) is derived from IPT, but is a briefer and more structured version that has demonstrated effectiveness in a variety of populations [24], including primary care outpatients [25–28], medically ill older adults aged 60 or more [29], patients with psychological distress post major physical trauma [30], women with breast cancer and their partners [31–34], men with prostate cancer and their supportive partners [35], patients with recent myocardial infarctions [36, 37], and women after miscarriage [38, 39]. IPC has been effectively delivered by trained health providers that are not mental health specialists, such as nurses practitioners [28] and doctors [25]. Mental health professionals have also effectively delivered IPC including psychiatric nurses [29, 30, 32–34], clinical psychologists [27, 30, 40], IPT-certified psychotherapists [38], psychiatric social workers [38], and residents in psychiatry with at least 2 years of clinical experience [27, 40]. However, IPC has never been task shifted to lay professionals with only a high school level education. Given the widespread shortage of mental health specialists in Brazil, the purpose of this trial was to determine whether IPC delivered by non-specialist community health workers could achieve comparable effectiveness to current available services by mental health specialists within the Brazilian public health system. To our knowledge, this is the first study to use IPC with a task shifting approach to train lay professionals in a low-resource setting.

## Methods

### Study design

This pragmatic Randomized Controlled Trial (RCT) sought to treat depressive disorders (current MDD or Dysthymia) according to American Psychiatric Association’s Diagnostic and Statistical Manual of Mental Disorders 4th Edition (DSM-IV). We sought to evaluate whether IPC, an abbreviated version of IPT with an abundant evidence base, could effectively be delivered by lay community health workers. The control group received Enhanced Treatment as usual (E-TAU), in which research psychologists optimized access to mental health resources available within the public health system.

### Setting

The RCT was conducted in the FHS Unidade Básica de Saúde Iaçapé, Sapopemba, district of São Paulo – Brazil between May 1, 2013 and April 30, 2015. All consecutive individuals attending routine visits were screened by the community health worker for eligibility based on the specified inclusion/exclusion criteria and randomly assigned to receive either IPC or E-TAU, using a parallel-group randomized controlled trial design [1:1]. The Institutional Review Board of the Federal University of São Paulo and the County Health Council of São Paulo city approved the study protocol. The trial was registered at Brazilian Clinical Trials, number RBR-5qhmb5 (trial url: http://www.ensaiosclinicos.gov.br/rg/RBR-5qhmb5/).

### Sample

Participation in the study was voluntary without financial compensation and written informed consent was obtained, in compliance with the Code of Ethics of the World Medical Association (Declaration of Helsinki) and the standards of the Review Board and granting agency. The inclusion criteria for the clinical trial were: (1) aged 18 or older; (2) positive screening for probable depressive disorder using the Zung scale [41] administered by community health workers with scale score confirmed by research psychologists; and (3) diagnosis by a research psychologist of current MDD or dysthymia using the Mini-International Neuropsychiatric Interview (MINI), a structured clinical diagnostic instrument based on the Diagnostic and Statistical Manual IV [42]. Exclusion criteria were: (1) ongoing treatment with antidepressants or psychotherapy; or (2) suicide risk evaluated by the MINI; or (3) current/previous episodes of mania, hypomania or current/previous psychotic symptoms, alcohol or psychoactive substance use disorder according to the MINI.

### Depression screening

Two authors (CTM and RFB) facilitated a 1-day training to 42 community health workers within the catchment area. We discussed the diagnosis criteria of MDD (DSM-IV) and practiced administration of the instrument Zung self-rating depression scale [41]. Although the scale can be self-administered, given the low literacy of the target population, community health workers were trained to administer all questions to recruit participants. The community health workers were employees of the County Health Council of São Paulo city, and there was no additional monetary compensation provided to screen participants.

### Interpersonal counseling (IPC)

IPC seeks to address patients’ current psychological problems and social within four interpersonal problem areas: prolonged grief, interpersonal disputes, role transitions and interpersonal deficits. Following the manual [28, 43], IPC comprised a 1-h session per week, with 3–4 sessions in total. Sessions were provided either at the clinic or in household visits, based on the individual’s preference. The community health workers facilitating IPC were not workers in the catchment area in which the patients resided to ensure confidentiality and prevent interference in their usual roles. We conducted a 3-day training to the 42 community health workers employed at the Health Unit, divided into three groups. Two of the authors (CTM and RFB, interpersonal therapists) facilitated the training using the Revised IPC Manual [43]. The training included research ethics and confidentiality, depression education with interactive activities, and role-playing of IPC techniques. Although all 42 community health workers had participated in the training, 20 were selected, according to motivation and empathy skills observed by the facilitators. They were supervised through the trial by the same trainers in 2 different groups in 2-h long twice a month supervision meetings. Supervisors were also available by telephone, mobile messages or email. These selected community health workers received a monetary compensation for each session completed.

### Enhanced treatment as usual (E-TAU)

FHS primary-care clinics usually have no mental health professionals employed onsite and referrals are required, however, this additional step often serves as a barrier for individuals to access treatment. We designed this arm to facilitate patients’ referral to specialized mental health care within the public system. Individuals randomized to E-TAU were provide case-management by off-site research psychologists funded by the study that were not trained in IPC. Research psychologists reported cases to FHS and facilitated referrals to specialized mental health care centers within the public system, where IPC is not provided, to receive either pharmacological or psychological treatment. The assigned research psychologist made 2–3 phone calls to the patient to check on the referral status and ensure follow-up. We considered E-TAU as received when a patient followed the task to complete the referral, even if there was a waiting list for treatment.

### Instruments

Research psychologists collected standard demographic information and administered the following instruments.

1. Pre-screening:

Zung Self-Rating Depression Scale [41]: A 20-item self-report questionnaire covering affective, psychological and somatic symptoms associated with depression. A total score ranges from 20 to 80, and we use a cutoff point of ≥45 as inclusion criteria, according to the validated Brazilian version [44].

*Mini-International Neuropsychiatric Interview* (MINI) [42]: A short semi-structured diagnostic interview based on DSM-IV criteria was used to confirm clinical diagnoses prior to the intervention. We used a version validated for use in Brazil [45, 46] and included the following modules: MDD, Dysthymia, Generalized Anxiety Disorder, Panic Disorder, Agoraphobia, Social Phobia and Post-Traumatic Stress Disorder.

2- Primary outcomes: (assessed at baseline and at 2-month follow-up visit)

*Health Questionnaire 9-item screen* (PHQ-9) [47]: A self-report measure of depression administered by the research psychologist to assess depressive symptoms over the past two weeks. Total scores on the scale range from 0 to 27 and recommended severity classifies people as having no (0–4), mild (5–9), moderate (10–14), moderately severe (15–19) or severe (20–27) depression. We used the Brazilian version translated and validated for use with primary care patients [48].

*Hamilton Depression Rating Scale* (HDRS-17) [49]: The 17-item version is a clinician-rated scale that determines the severity of each symptom during the past week. Total scores range from 0 to 52, and the American Psychiatric Association’s Handbook of Psychiatric Measures [50] defines its grades of severity as mild depression (8–13), moderate depression (14–18), severe depression (19–22), and very severe depression (≥23). We used the translated Brazilian version [51].

The primary outcome of this study was the reduction of scores in PHQ-9 [47] and HDRS-17 [49]. Full remission of the depressive disorder was defined as an HDRS-17 score of 8 or less [52]. Following Hollon et al. [53], HDRS-17 scores ≤12 were considered to meet criteria for partial remission.

3- Secondary outcomes: (assessed at baseline and at 2-month follow-up visit)

*The Self-Reporting Questionnaire* (SRQ-20) [54] was specifically developed by WHO to identify minor psychiatric morbidity in primary care and community settings in developing countries. It includes 20 dichotomous items covering depressive, anxiety and somatic symptoms. We used a cutoff of ≥8 for positive cases according to the translated Brazilian version [55].

*The Clinical Global Impression instrument* (CGI) [56] provides an overall score of the clinician’s view of the patient’s symptoms, behavior and functioning following a seven-point scale for one question. It ranges from 1 (normal) to 7 (extremely severe symptoms).

For secondary outcomes, we analyzed changes in the scores of the SRQ-20 [54, 55] and CGI [56].

### Randomization

Randomization (allocation ratio 1:1) was stratified by gender, age (17–34 vs. ≥35), and depression severity (Zung score 45–59 vs. ≥60). A statistician not involved in the recruitment process carried out the randomization using a computer algorithm based on Aitchison’s compositional distance [57]. Only the research assistant knew the group allocation. Evaluators at follow up were blinded to which intervention was received.

### Statistical analyses

We performed the analyses using the intention-to-treat approach. Descriptive statistics included mean and standard deviation (SD) for continuous variables, and frequencies for categorical or ordinal variables. Comparisons between the two groups (IPC vs. E-TAU) were performed using Student’s t-test for continuous variables, and cross-tabulation with χ2 test for categorical or ordinal variables. Statistical Package for the Social Sciences (SPSS) version 23 [58] was used to perform these analyses. Given the relatively small sample size, we tested group differences on primary and secondary outcomes using Latent Growth Model (LGM) and Bayesian estimator [59]. Goodness-of-fit of the models was evaluated using the Posterior Predictive *P*-values (PPP), which should be close to 0.5. Mplus 7.4 was used to perform these analyses.

When performing LGM, Mplus estimates a slope representing the mean change over time in the outcome. Group differences were estimated by regressing the slope on a binary variable identifying the two groups IPC vs. E-TAU (0/1). The effect size was calculated using the Independent-Groups Pretest-Posttest formula described in Feingold [60]. Later, we performed a per-protocol analysis for primary outcomes, i. e. involving only those patients who completed the intervention originally assigned.

## Results

**Fig. 1:**
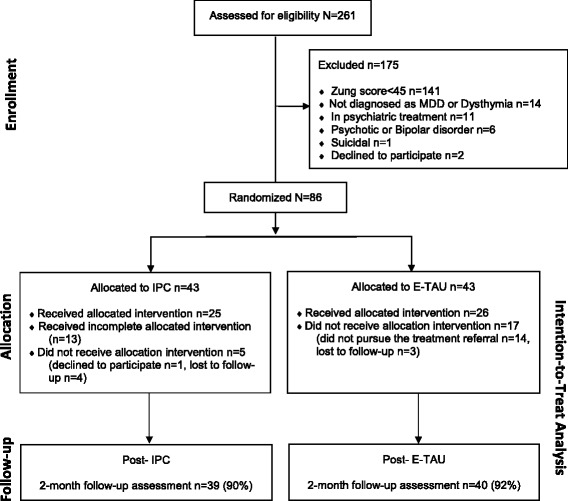
CONSORT flow diagram. IPC – Interpersonal Counseling group, E-TAU – Enhanced Treatment as Usual group

A diagram of participant flow is depicted in Fig. 1. A total of 261 patients were recruited, 175 excluded for not meeting eligibility criteria (*n* = 173) or declined to participate (*n* = 2) and 86 patients were randomized to IPC (*n* = 43) or E-TAU group (*n* = 43). Of the 43 patients that were selected to receive IPC, 38 received a mean 2.26 (±1.26) sessions. Twenty-five (58.14%) received three or four sessions. Thirteen (30.23%) patients attended one or two sessions. Five (11.63%) did not attend any sessions: 1 declined (2.33%) and 4 were lost to follow-up (*n* = 4; 9.30%). Among the 43 patients selected for E-TAU, 26 (60.50%) received the allocated intervention: 13 (30.25%) engaged in treatment (pharmacotherapy or psychological) available in the public system and 13 (30.25%) were on waiting lists for treatment. Seventeen (39.50%) did not receive any intervention, either because they did not pursue the treatment referral (*n* = 14; 32.56%) or were considered lost to follow up (*n* = 3; 6.94).

Demographic characteristics of the study population are described in Table 1. The two groups had similar clinical profiles at baseline (see Table 2). No differences in gender, age, ethnicity, education, religion and social-economical class were observed between the IPC and E-TAU group.

**Table 1:** Demographic characteristics of the study population

	Total	IPC group	E-TAU group		
	*N* = 86	*n* = 43	*n* = 43	Statistics	*p*
Age, years: mean (s.d.)	43.84 (14.76)	46.42 (15.22)	41.26 (13.99)	*t* = 1.64	.11
Gender, female: n (%)	81 (94.20)	40 (93.00)	41 (95.30)	ϰ^2^ = 0.21	.65
Race/ethnicity: n (%)				ϰ^2^ = 4.22	.24
African American/Black	8 (9.30)	4 (9.30)	4 (9.30)		
White/Caucasian	36 (41.90)	19 (44.20)	17 (39.50)		
Biracial/Multiracial	38 (44.20)	20 (46.50)	18 (41.90)		
Other	4 (4.70)	0 (0.00)	4 (9.30)		
Marital status: n (%)				ϰ^2^ = 3.11	.08
Not partnered	34 (39.50)	13 (30.20)	21 (48.80)		
Number of children:				ϰ^2^ = 2.83	.59
0	25 (29.10)	11 (25.60)	14 (32.60)		
1	31 (36.00)	18 (41.90)	13 (30.20)		
2	24 (27.90)	10 (23.30)	14 (32.60)		
≥3	6 (7.00)	4 (9.30)	2 (4.60)		
Education: n (%)				ϰ^2^ = 4.16	.13
< High school degree	51 (59.30)	30 (69.80)	21 (48.80)		
High school degree or equivalent	28 (32.60)	11 (25.60)	17 (39.50)		
Education beyond high school degree	7 (8.10)	2 (4.70)	5 (11.60)		
Religion: n (%)				ϰ^2^ = 2.11	.55
Catholic	32 (37.20)	16 (37.20)	16 (37.20)		
Protestant	35 (40.70)	18 (41.90)	17 (39.50)		
Other	5 (5.80)	1 (2.30)	4 (9.30)		
None	14 (16.30)	8 (18.60)	6 (14.00)		
Socio-economical class^a^: n (%)				ϰ^2^ = 6.18	.19
A1/A2	2 (2.30)	2 (4.70)	0 (0.00)		
B1/B2	21 (24.40)	12 (27.90)	9 (20.90)		
C1/C2	48 (55.80)	19 (44.20)	29 (67.50)		
D	9 (10.50)	6 (14.00)	3 (7.00)		
E	6 (7.00)	4 (9.30)	2 (4.70)		
Household monthly income^b^ (US$): mean (s.d.)	687.74 (603.79)	785.10 (746.07)	595.37 (417.37)	*t* = 1.38	.17

^a^ABIPEME Brazilian socio-economical class classification

^b^Conversion R$ 1.00 (reais) = US$ 3.5 (dollar), August 2015.

**Table 2:** Clinical characteristics of the study population at baseline

	Total	IPC group	E-TAU group		
	*N* = 86	*n* = 43	*n* = 43	Statistics	*p*
Zung Total: mean (s.d.)	53.47 (6.69)	54.28 (7.49)	52.65 (5.75)	*t* = 1.28	.26
HDRS-17 severity: N (%)				ϰ^2^ = 6.15	.19
Normal (0–7)	3 (3.50)	0 (0.00)	3 (7.00)		
Mild (8–13)	18 (20.90)	9 (50.00)	9 (50.00)		
Moderate (14–18)	30 (34.90)	13 (30.20)	17 (39.50)		
Severe (19–22)	21 (24.40)	11 (25.60)	10 (23.30)		
Very severe (≥23)	14 (16.30)	10 (23.30)	4 (9.30)		
HDRS-17 Total score: mean (s.d.)	17.13 (5.79)	18.30 (5.83)	15.93 (5.56)	*t* = 3.69	.06
PHQ-9 severity: N (%)				ϰ^2^ = 2.93	.57
Minimal (0–4)	1 (1.20)	0 (0.00)	1 (2.30)		
Mild (5–9)	6 (7.00)	3 (7.00)	3 (7.00)		
Moderate (10–14)	21 (24.40)	10 (23.30)	11 (25.60)		
Moderately severe (15–19)	32 (37.20)	14 (32.60)	18 (41.90)		
Severe (20–27)	26 (30.20)	16 (37.20)	10 (23.30)		
PHQ-9 Total score: mean (s.d.)	16.80 (5.06)	17.58 (5.16)	16.02 (4.89)	*t* = 2.07	.16
SRQ: mean (s.d.)	13.55 (3.46)	13.79 (3.52)	13.30 (3.41)	*t* = 0.43	.52
CGI: mean (s.d.)	4.70 (0.77)	4.53 (0.67)	4.86 (0.83)	*t* = 1.92	.06
MINI (DSM-IV and ICD-10): N (%)					
Depressive Disorder	86 (100.00)	43 (100.00)	43 (100.00)	ϰ^2^ = 0.00	1.0
Major Depressive Episode, current	81 (94.20)	43 (100.00)	38 (88.40)	ϰ^2^ = 5.31	**.02**
MDE, recurrent	30 (34.90)	15 (34.90)	15 (34.90)	ϰ^2^ = 0.00	1.0
MDE, single episode	51 (59.30)	28 (65.10)	23 (53.50)	ϰ^2^ = 1.20	.27
Dysthymia	6 (7.00)	1 (2.30)	5 (11.60)	ϰ^2^ = 2.87	.09
MDD + Dysthymia	1 (1.20)	1 (2.30)	0 (0.00)	ϰ^2^ = 1.01	.31
MDE with melancholic features	48 (55.80)	26 (60.50)	22 (51.20)	ϰ^2^ = 0.75	.39
Comorbidity with DD	28 (32.60)	12 (27.90)	16 (37.20)	ϰ^2^ = 0.85	.36
Generalized Anxiety Disorder	7 (8.10)	3 (7.00)	4 (9.30)	ϰ^2^ = 0.16	.69
Panic Disorder	5 (5.80)	2 (4.70)	3 (7.00)	ϰ^2^ = 0.21	.64
Agoraphobia	11 (12.80)	5 (11.60)	6 (14.00)	ϰ^2^ = 0.10	.75
Social Phobia	6 (7.00)	3 (7.00)	3 (7.00)	ϰ^2^ = 0.00	1.0
Obsessive Compulsive Disorder	1 (1.20)	1 (2.30)	0 (0.00)	ϰ^2^ = 1.01	.31
Post-Traumatic Stress Disorder	6 (7.00)	2 (4.70)	4 (9.30)	ϰ^2^ = 0.72	.40

*HDRS-17* – 17-item Hamilton Depression Rating Scale, *PHQ-9* – Patient Health Questionnaire, *SRQ-20* – Self-Reporting Questionnaire, *CGI* – Clinical Global Impression instrument, *MINI* − Mini-International Neuropsychiatric Interview, *MDE* – Major Depressive Episode; *IPC* – Interpersonal Counseling group, *E-TAU* – Enhanced Treatment as Usual group

For the total sample (*N* = 86), the mean total score of PHQ-9 at baseline was 16.80 (±5.06) and the mean total score of HDRS-17 was 17.13 (±5.79). All patients included in the study met diagnostic criteria for a depressive disorder: current Major Depressive Episode (*n* = 81; 94.20%) or Dysthymia (*n* = 6; 7.00%), accordingly to clinical assessment using the MINI. Only one patient met both diagnostic criteria. Thirty (34.90%) had recurrent Major Depressive Episode and 51 (59.30%) presented the first single Major Depressive Episode. Forty-eight (55.80%) met criteria for MDD with melancholic features. Twenty-eight (32.60%) had at least one psychiatric comorbidity with depressive disorder such as Generalized Anxiety Disorder, Panic Disorder, Agoraphobia, Social Phobia, Obsessive Compulsive Disorder or Post-Traumatic Stress Disorder. IPC group had higher frequency of current Major Depressive Episode than E-TAU group (100.00% vs. 88.40%; ϰ2 = 5.31; *p* = 0.02), which was the only clinical profile difference at baseline.

The fit of the four LGMs was good (PPP close to .50; see Table 3). The results of the analysis (LGM) did not show statistically significant differences between the two groups (IPC vs. E-TAU) (see Table 3) for primary and secondary outcomes. However, the differences approached statistical significance (i.e. *p* < .10) that can be interpreted as trends towards medium effect sizes. Overall, both groups showed significant improvement on depressive symptoms (HDRS-17 and PHQ-9), with lower SRQ-20 and CGI score after 2 months.

**Table 3:** Intention-to-treat analysis. Mean scores (SD) by allocated intervention and observation time

Outcome	Baseline IPC (*n* = 43) E-TAU (*n* = 43)	2-month IPC (*n* = 39) E-TAU (*n* = 40)	PPP	∆ Time by group (beta)	*p*	CI (95%)^a^	∆ Time (beta)	*p*	CI (95%)^a^	Effect size
HDRS-17										
IPC	18.30 (5.83)	13.31 (7.08)	.46	2.23	.08	−.78 to 5.24	−1.23	.05	−2.71 to −.25	0.08
E-TAU	15.93 (5.56)	13.03 (6.56)								
PHQ-9										
IPC	17.58 (5.16)	11.36 (6.98)	.45	.99	.09	−.31 to .02	−2.56	<.001	−3.29 to −1.84	0.10
E-TAU	16.02 (4.89)	11.83 (5.67)								
SRQ-20										
IPC	13.79 (3.52)	9.67 (5.46)	.46	.74	.09	−0.34 to 1.84	−1.70	<.001	−2.25 to −1.14	
E-TAU	13.30 (3.41)	10.65 (4.40)								
CGI										
IPC	4.86 (0.83)	3.21 (1.22)	.46	.12	.21	−.19 to .42	−.76	<.001	−.92 to −.61	
E-TAU	4.53 (0.67)	3.12 (1.21)								

^a^Student’s t-test

HDRS-17 – 17-item Hamilton Depression Rating Scale, PHQ-9 – Patient Health Questionnaire, SRQ-20 – Self-Reporting Questionnaire,

CGI – Clinical Global Impression instrument, IPC – Interpersonal Counseling group, E-TAU – Enhanced Treatment as Usual group

∆ Time by group = change overtime in between groups (IPC vs. E-TAU)

∆ Time = change overtime within group

*PPP* = posterior predictive *p*-value

In the IPC group, 11 (28.21%) participants achieved complete remission (HDRS-17 score ≤ 8) compared to 9 (22.50%) participants of E-TAU group, with no difference between the two groups (ϰ2 = 0.34; *p* = 0.56). Partial remission (HDRS-17 score ≤ 12) was achieved by 16 (41.03%) from IPC group vs. 17 (42.50%) from E-TAU group, with no difference between the two groups (ϰ2 = 0.02; *p* = 0.89). In IPC group, 10 (25.64%) participants achieved response to treatment (50% reduction of symptoms) and 6 (15.38%) from E-TAU group, with no difference between the two groups (ϰ2 = 1.26; *p* = 0.26).

**Fig. 2:**
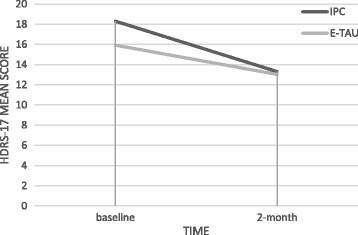
Intention-to-treat analysis. Mean scores of HDRS-17 by allocated intervention and observation time. HDRS-17 – 17-item Hamilton Depression Rating Scale, IPC – Interpersonal Counseling group, E-TAU – Enhanced Treatment as Usual group. Change overtime in between groups: Beta 2.23; *p* = 0.08; CI (95%) = −0.78 to 5.24. Change overtime within group: Beta −1.23; *p* = 0.05; CI (95%) = −2.71 to −0.25

**Fig. 3:**
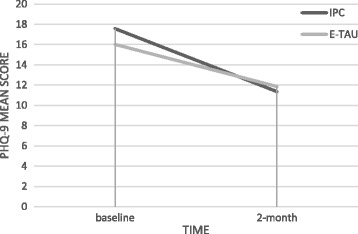
Intention-to-treat analysis. Mean scores of PHQ-9 by allocated intervention and observation time. PHQ-9 – Patient Health Questionnaire, IPC – Interpersonal Counseling group, E-TAU – Enhanced Treatment as Usual group Change overtime in between groups: Beta 0.99; *p* = 0.09; CI (95%) = −0.31 to 0.02. Change overtime within group: Beta −2.56; *p* = <0.001; CI (95%) = −3.29 to −1.84

Primary outcomes are graphically shown in Figs. 2 and 3.

**Fig. 4:**
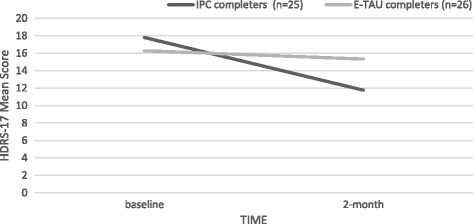
Per-protocol analysis. Mean scores of HDRS-17 of completers by allocated intervention and observation time. HDRS-17 – 17-item Hamilton Depression Rating Scale, IPC – Interpersonal Counseling group, E-TAU – Enhanced Treatment as Usual group. Change overtime in between groups: Beta 2.68; *p* = 0.003; CI (95%) = 0.86 to 4.46. Change overtime within group: Beta −1.71; *p* = 0.001; CI (95%) = −2.69 to −0.73

To investigate these results further we applied a per-protocol analysis for those patients who completed the intervention as intended (IPC *n* = 25 vs. E-TAU *n* = 26). Primary outcomes were analyzed. Regarding the HDRS-17, the fit of the LGM was good (PPP = 0.42), and the results showed a statistically significant difference between the two groups (IPC vs. E-TAU), see Table 4 and Fig. 4. Regarding the PHQ-9 scale, the fit of the LGM was good (PPP = .42), and the results did not show a statistically significant difference between the two groups (IPC vs. E-TAU), see Table 4. Change over time in all groups showed significant improvement on depressive symptoms based on HDRS-17 and PHQ-9 scores.

**Table 4:** Per-protocol analysis. Mean scores (SD) by allocated intervention and observation time

Outcome	Baseline IPC (*n* = 25) E-TAU (*n* = 26)	2-month IPC (*n* = 25) E-TAU (*n* = 25)	PPP	∆ Time by group (beta)	*p*	CI (95%)^a^	∆ Time (beta)	*p*	CI (95%)^a^	Effect size
HDRS-17										
IPC	17.80 (5.66)	11.76 (6.27)	.42	2.68	.00	.86 to 4.46	−1.71	.00	−2.69 to −.73	0.14
E-TAU	16.28 (5.89)	15.32 (6.10)								
PHQ-9										
IPC	17.00 (5.83)	12.29 (5.61)	.42	−.61	.29	−2.79 to 1.59	−2.56	<.001	−3.62 to −1.49	0.25
E-TAU	16.65 (4.92)	10.86 (7.06)								

^a^Student’s t-test

HDRS-17 – 17-item Hamilton Depression Rating Scale, PHQ-9 – Patient Health Questionnaire, SRQ-20 – Self-Reporting Questionnaire,

CGI – Clinical Global Impression instrument, IPC – Interpersonal Counseling group, E-TAU – Enhanced Treatment as Usual group

∆ Time by group = change overtime in between groups (IPC vs. E-TAU)

∆ Time = change overtime within group

*PPP* = posterior predictive p-val

## Discussion

This trial demonstrates that training community health workers using a task shifting approach to improve MDD symptoms is not only acceptable and feasible, but can achieve comparable positive results to an optimized treatment as usual with case management by psychologists. This is consistent with other studies suggesting the potential effectiveness of community health workers’ psychosocial and psychological interventions [19, 20]. Whereas intention-to-treat analysis showed significant improvement on symptoms for both groups over 2 months, without significant differences between them, per-protocol analysis resulted in a significantly better outcome for the IPC group. Providing mental health services, including evidenced-based interventions, in primary care clinics or systems can be done in many different ways. Traditionally, many clinics opt to refer patients to specialists as needed. However, the implementation is challenging because it is not always feasible to employ on-site care managers to enhance referral, so training on-site providers in evidenced-based interventions could be a better option, also in terms of acceptability. IPC group had better acceptability with only 1 (2.33%) patient declining to participate versus 14 (32.56%) that did not pursue the treatment referral in the E-TAU group.

To date, IPC has been studied in diverse populations with highly trained health workers as providers [24]. The only large IPC trial with a sample of 287 patients showed robust results when IPC was delivered by clinical psychologists or psychiatric residents with a minimum of 2 years of clinical experience [26, 27]. Our study demonstrates that task shifting IPC to lay community health workers can achieve comparable results to E-TAU. Although the research psychologists from the E-TAU group did not offer any standardized treatment, their engagement increased clinical attention, an effect not present in routine clinical care, which could explain positive outcomes in the control group.

Despite finding low remission rates in HDRS-17 (28.21% in IPC group and 22.50% in E-TAU group), it is known that only about a third of patients with an episode of MDD may remit with a given treatment [61]. Although IPC is proven to be more efficacious than selective serotonin reuptake inhibitors (SSRIs) in primary care patients with mild to moderate depression [27], in our sample 40.70% exhibited severe to very severe depression according to HDRS-17 (≥19) and also responded to treatment. This high MDD severity was not expected for a primary care setting, nevertheless it suggests possible bias for our intervention outcomes since the slightly lower remission rates in HDRS-17 and other overall results may be because of a particularly symptomatic population. According to our study design, the patients were recruited in the community because most of them would not seek care spontaneously at the FHS clinic. The severity of symptoms among patients recruited also calls attention to the needs for depression screening and earlier treatment.

When we further investigated using a per-protocol analysis, including only completers of IPC and E-TAU, we found a statistically significant difference with greater improvement for the IPC group on HDRS-17, but not based on PHQ-9 score. We opted to use the PHQ-9 as it is faster to administer, less complicated to score and could be applied to a broader range of practice settings, even though HDRS-17 has been the criterion standard outcome measure used in clinical trials [62, 63].

At the end of the study, 18 community health workers, of the 20 that were selected, had actively participated throughout. Many community health workers reported empowerment and better understanding of mental health issues after receiving the training and following patients in their daily lives. Therefore, it’s possible that training community health workers in mental health could also be a way to enhance prevention and reduce mental health stigma in the community [21, 64].

We acknowledge some limitations of the present study. First, this was a pilot study with a small sample and effect size, where patients were recruited from a single FHS catchment area. Also, whereas inclusion criteria and randomization were based on depression symptoms from the Zung screening scale, outcomes were measured using different scales. We also had low compliance rates, with 58.14% completing the 3–4 sessions of IPC and 60.50% completing referral based on E-TAU protocols, which could have been related to cultural stigma, religious beliefs or socioeconomically vulnerability in this population, or not being accustomed to valuing mental health beyond other subsistence needs. That said, low adherence is commonly observed in LMIC settings, and studies have shown that fewer than 40% of adults entering psychotherapy ever receive more than 3 to 5 sessions [24]. Our results support the feasibility, acceptability, and potential effectiveness of a short treatment provided by community health workers compared to traditional psychotherapy.

## Conclusions

Immediate action is required to reach out to individuals with depression in primary care. Using an intention-to-treat analysis, both arms, training on-site community health workers in IPC or enhancing referral, were effective methods to improve depressive symptoms. Per-protocol analysis resulted in a significantly better outcome for the IPC group. The results point to the need for larger trials with longer follow-up period to verify the long-term effectiveness of IPC in primary care. This could be a key strategy for closing the mental health treatment gap in the Brazilian FHS and other LMICs.

## Abbreviations

CGI: Clinical Global Impression instrument
DSM-IV: Statistical Manual of Mental Disorders 4th Edition
E-TAU: Enhanced Treatment as Usual
FHS: Family Health Strategy
HDRS: Hamilton Depression Rating Scale
ICD-10: International Classification of Diseases 10th Revision
IPC: Interpersonal Counseling
IPT: Interpersonal Psychotherapy
LGM: Latent Growth Model
LMIC: Low and Middle-Income Countries
MDD: Major Depressive Disorder
MINI: Mini-International Neuropsychiatric Interview
PHQ: Patient Health Questionaire
PPP: Posterior Predictive *P*-values
RCT: Randomized Controlled Trial
SD: Standard Deviation
SPSS: Statistical Package for the Social Sciences
SRQ: Self-Report Questionnaire
SSRIs: Selective Serotonin Reuptake Inhibitors
WHO: World Health Organization
